# Analysis of Ferric Protoporphyrin IX Effects on Human Platelets: Hematin Is a More Potent Agonist than Hemin

**DOI:** 10.3390/cells14040255

**Published:** 2025-02-11

**Authors:** Diana M. Mikhailova, Julia Sudnitsyna, Polina Kovgan, Lidia Naida, Alexandra Kharazova, Igor Mindukshev, Stepan Gambaryan

**Affiliations:** 1 Sechenov Institute of Evolutionary Physiology and Biochemistry, Russian Academy of Sciences, 44 Thorez Ave., 194223 Saint Petersburg, Russia; mikhailowa.dm@gmail.com (D.M.M.); julia.sudnitsyna@uni-wuerzburg.de (J.S.); kovgan.pe@gmail.com (P.K.); nayda.lidiyaa@gmail.com (L.N.); iv_mindukshev@mail.ru (I.M.); 2 Department of Cytology and Histology, Saint Petersburg State University, 7/9 Universitetskaya Emb., 199034 Saint Petersburg, Russia; akharazova@gmail.com; 3 Institute of Biomedical Systems and Biotechnologies, Peter the Great Saint Petersburg Polytechnic University, 195251 Saint Petersburg, Russia

**Keywords:** platelets, hematin, hemin, aggregation, shape change, albumin, laser diffraction

## Abstract

Hemolysis during severe diseases (malaria, hemorrhagic stroke, sickle cell disease, etc.) and blood transfusion induces the release of free hemoglobin, which degrades to highly reactive and toxic compounds—hemin and hematin. Oxidized heme derivatives induce platelet activation, aggregation, and degranulation, leading to prothrombotic and inflammatory events. In the present study, we showed that hematin is a more potent agonist of platelet activation than hemin, and using several methods, including the original laser diffraction method, flow cytometry, and confocal microscopy, we demonstrated that hematin at low doses induces platelet activation and aggregation without reducing cell viability and affecting calcium efflux. On the contrary, hematin at high concentrations triggered phosphatidylserine exposure, severe loss of platelet viability, and calcium dysregulation, which was not inhibited by cGMP/PKG and cAMP/PKA pathways. Additionally, we showed that albumin could initiate disaggregation processes in hematin-activated platelets.

## 1. Introduction

Intravascular hemolysis is widely associated with severe pathologies such as hemolytic uremic syndrome, autoimmune hemolytic anemia, sickle cell disease, hemorrhagic stroke, and malaria and may also occur following blood transfusion [1,2,3,4]. Hemolysis is associated with thrombosis and vascular dysfunction as the hemolytic products formed overwhelm the body’s scavenging systems, provoking unwanted endothelial cell and platelet activation and severe inflammation [5,6]. During hemolysis, red blood cells release cell-free hemoglobin, which disintegrates into free heme, which in turn can be oxidized to such hemolytic products as hemin (ferric (Fe^3+^) protoporphyrin (PP) IX liganded with Cl^−^) or hematin (ferric (Fe^3+^) PP IX liganded with OH^−^), which are often referred to as hemin, neglecting the individual structural and pathophysiological features of both compounds [7].

In physiological conditions, free hemin/hematin is scavenged by albumin and hemopexin and subsequently catabolized by hemoxygenase-1 to carbon monoxide, biliverdin, and iron (Fe^2+^) [8]. However, both acute and chronic hemolysis deplete the body’s scavenging systems, leading to elevated concentrations of heme derivatives in the blood. For example, systemic heme concentrations of up to 50 μM have been observed in plasma samples from hemolytic patients [9]. High hemin/hematin concentrations provoke endothelial cell and platelet activation, platelet hypercoagulability, thrombocytopenia, aggravated erythrocyte lysis, and neutrophil extracellular trap formation, which consequently lead to severe inflammation, thrombosis, and organ damage [3,5,10,11]. Thus, secondary brain injury in hemorrhagic stroke strongly correlates with increased concentrations of heme derivatives due to the toxic effects of iron formed during the decomposition of ferric protoporphyrin IX [12], therefore it is essential to find strategies to improve the organism’s capacity for scavenging free heme and heme derivatives in pathological complications.

Several studies have investigated the development of thrombotic complications caused by hemin/hematin [13,14]. For instance, in vulnerable atherosclerotic plaques, intraplaque hemorrhage (IPH) leads to hemolysis followed by free hemin release, triggering thromboischemic clinical events [15]. Recently, it was revealed that hemin could be a potential ligand for C-type lectin-like receptor 2 (CLEC-2) and glycoprotein VI (GPVI) platelet receptors, resulting in platelet activation, degranulation, and aggregation [16,17]. Hemin was also demonstrated to bind to GPIbα, which may potentially regulate platelet clearance by macrophages in hemolytic disease [18]. Noteworthy, hemin-induced platelet activation was not affected by classical platelet inhibitors such as cyclooxygenase inhibitor (indomethacin) or P2Y12 inhibitor (cangrelor), indicating a unique mechanism of hemin-triggered platelet responses [16]. High concentrations of heme derivatives were shown to trigger platelet ferroptosis, an intracellular iron-mediated cell death that differs from classical apoptosis and supposedly induced platelet agglutination [19,20]. Interestingly, all hemin-dependent alterations were significantly attenuated in the presence of pro-protein convertase furin inhibition [21], thus connecting the cellular hemostasis with systemic cardiovascular/natriuretic peptide system [22] and suggesting the presence of a unique specific to platelets hemin-triggered transformation pathway.

Despite many studies, the specific mechanisms involved in hemin-induced platelet activation remain unclear. For instance, the deletion of CLEC-2 in mice did not inhibit platelet shape change, suggesting the possible involvement of other receptors in facilitating hemin-induced platelet activation [16]. The role of elevated hemin concentrations in possible platelet agglutination is still uncertain. At the same time, conventional antiplatelet therapy strategies have proven ineffective in preventing hemin-induced platelet activation and thrombus formation. Therefore, a detailed analysis of the potential protective effects of albumin on platelets is of particular interest. Another critical question is whether the effects of hematin on human platelets would exhibit the same pattern as hemin and if there are differences between the mechanisms of action of both heme-containing compounds. These data could be of particular importance for malaria or porphyria research as when well-studied hemin is constantly produced in the human body during the reactions of Hb autooxidation, either spontaneous or disease-triggered, then hematin is formed predominantly during malaria, gastric disorders, or supplemented during porphyria treatment [23,24].

In this study, we showed that hematin exhibits higher reactivity towards platelet transformation compared to hemin. Hematin induced platelet activation and aggregation at low doses (5 μM) and did not induce agglutination at higher concentrations (30 μM). Hematin-induced activation and aggregation at high doses were accompanied by a persistent increase in intracellular calcium, phosphatidylserine exposure, and a significant decrease in cell viability, indicating the destructive effect of hematin on human platelets. As a scavenger of free hematin, albumin triggered platelet disaggregation and potentially stabilized platelet morphology in the presence of low hematin doses. These findings have potential clinical implications, especially for conditions such as malaria, porphyria, etc., where systemic hematin predominates over hemin. A deeper understanding of these mechanisms could improve the development of therapeutic strategies for managing platelet dysfunction and thrombotic complications in the aforementioned pathologies.

## 2. Materials and Methods

### 2.1. Ethics Approval

The research was conducted according to the Declaration of Helsinki and approved by the Ethical Committee of the Sechenov Institute of Evolutionary Physiology and Biochemistry of the Russian Academy of Sciences. Human blood was obtained from healthy donors by venipuncture after signing the written consent (protocol no. 03–02 from 28 February 2024).

### 2.2. Reagents and Working Buffers

Hemin, albumin, Calcein-AM (C-AM, calcein), sodium nitroprusside (SNP), Human Albumin, iloprost, mepacrine, and working buffer components (HEPES, NaCl, KCl, MgCl_2_, D-glucose, EGTA, CaCl_2_) were purchased from Sigma-Aldrich (Darmstadt, Germany). Fluo-3-AM was obtained from Invitrogen (Carlsbad, CA, USA); thrombin was purchased from Roche (Mannheim, Germany); fibrinogen-Alexa-Fluor 647—from Molecular Probes (Göttingen, Germany); PE-conjugated CD62P, CD41, Annexin-V—from BD Bioscience (Heidelberg, Germany); ReoPro—from Lilly Deutschland GmbH (Giessen, Germany) and a kind gift from Prof. Dr. Mikhail Panteleev (CTP PCP RAS, Moscow, Russia), including collagen related peptide (CRP) were used.

To evaluate the effects of hemin/hematin on platelet transformations, the following working buffers were used: CGS buffer: 120 mM sodium chloride, 12.9 mM trisodium citrate, 10 mM D-glucose, pH 6.5; HEPES buffer: 150 mM sodium chloride, 3 mM potassium chloride, 1 mM magnesium chloride, 5 mM D-glucose, 10 mM HEPES, pH 7.4. Additionally, 1.5 mM CaCl_2_ was added to platelet suspension for flow cytometry experiments.

The osmolality of the buffers (300 mOsm/kg H_2_O) was controlled using a cryoscopic osmometer Osmomat 030 (Gonotec GmbH, Berlin, Germany).

### 2.3. Human Platelets Preparation

Human platelets were obtained as described previously [25]. In brief, venous blood was collected by caudal venipuncture in Citrate 9NC (0.106 mol/L/3.2%) S-monovette tubes (Sarstedt, Nümbrecht, Germany) with an addition of 2 mM EGTA and centrifuged at 300× *g* (centrifuge ELMI-50CM, Elmi, Riga, Latvia) for 8 min at RT. Subsequently, the supernatant containing plasma and platelets (platelet-rich plasma, PRP) was resuspended in CGS buffer and centrifuged at 2400 RPM for 4 min at RT. The resulting platelet pellets were resuspended in HEPES buffer and rested at RT until the experiment for 20 min. To monitor the platelet count and parameters, the Medonic-M20 hematological counter (Boule Medical A.B., Stockholm, Sweden) was used.

### 2.4. Hematin and Hemin Preparation

Hematin and hemin stock solutions were prepared according to [26,27]. Briefly, hemin powder was dissolved in DMSO to prepare a 1 mM hemin stock solution. To prepare a 1 mM hematin stock solution, hemin powder was dissolved in 20 mM NaOH. The concentrations of hematin and hemin were determined and controlled using the molar extinction method according to the Beer-Lambert equation using the compounds’ millimolar extinction coefficient at two different wavelengths (385 nm and 342 nm, respectively) [27]. Spectra were registered using a spectrophotometer (Spectroscopic Systems LTD, Moscow, Russia). For each experiment, hematin or hemin solutions were freshly prepared from powder and stored in the dark at 4 °C.

### 2.5. Analysis of Hematin- and Hemin-Induced Activation, Aggregation, and Disaggregation Using the Laser Diffraction Method

Hematin- and hemin-induced platelet activation and aggregation were analyzed by the laser diffraction method (laser microparticle analyzer LaSca-TM, BioMedSystems Ltd., Saint Petersburg, Russia) described in detail in [28]. Shortly, the laser beam (650 nm) passed through the platelet resuspended in HEPES buffer with 1.5 mM Ca^2+^ (2 × 10^7^ cell/mL final concentration) in the cuvette with continuous stirring (1200 rpm, 37 °C). The original laser diffraction particle analyzer constantly registers the buffer absorbance (at 0 degrees) and in our experiments, the differences after the addition of hematin (30 µM) were less than 1%.

The platelet shape change was characterized by an increase in the light scatter intensity (LSI) at the scattering angle of 12°. The platelet aggregation was characterized by the LSI increase at the scattering angle of 1° with a simultaneous LSI decrease at the scattering angle of 12°. The platelet disaggregation was characterized by a decline of LSI at 1° after reaching the point of 100% Aggregation. The following functional indices were used to describe quantitatively platelet responses to agonists: the initial velocity of aggregation (V*_aggregation_*) determined at 20 s after the agonist supplementation; the velocity of platelet shape change (V*_shape_*) assessed upon initiation of a shape change. The disaggregation percent was calculated as a decrease in the maximum LSI signal (taken as 100% of aggregation). For a detailed description, see Supplementary Figure S1.

### 2.6. Analysis of Platelet Ca^2+^ Mobilization

To analyze hematin-induced changes in intracellular Ca^2+^ concentration ([Ca^2+^]_i_), the upgraded laser microparticle analyzer LaSca-TMF equipped with 488 nm laser and FL1 fluorescence detector (527 nm) (BioMedSystems Ltd., Saint Petersburg, Russia) was used. The method is described in detail in [29]. PRP was incubated with Fluo-3-AM (10 µM, 60 min, RT) in the dark and then was centrifuged at 2400 RPM for 4 min, and platelet pellets were resuspended in HEPES buffer (2 × 10^7^ cell/mL final concentration). Intracellular Fluo-3 was excited at 488 nm, and the emission was registered at 527 nm (FL1). The area under the curve (AUC*_Ca_*) was calculated to characterize hematin-induced [Ca^2+^]_i_ changes in platelets (Supplementary Figure S2).

### 2.7. Flow Cytometry Analysis

Hematin- and hemin-induced platelet activation was analyzed by flow cytometry using the CytoFLEX flow cytometer (Beckman Coulter, Brea, CA, USA) with an analysis of not less than 15.000 events. Platelets were gated according to the CD41 positive events (Supplementary Figure S5A) according to [30].

#### 2.7.1. Analysis of Platelet aIIbβ3 Integrin Activation

To analyze platelet αIIbβ3 integrin activation, Fibrinogen-Alexa-Fluor 647 binding was used. Fibrinogen (final concentration 15 μg/mL) was added to washed platelets (2 × 10^7^ cell/mL final concentration), and cells were incubated at 37 °C for 30 min. Next, after the addition of agonists (hematin 5 μM, 30 μM, or thrombin 0.05 U/mL as a positive control), the samples were incubated at 37 °C for an additional 2 min. Finally, the reaction was stopped by PBS (1:40), and the median fluorescence intensity (MFI) was registered at the FL4 channel.

#### 2.7.2. Analysis of Alpha and Dense Granule Secretion

CD62P binding was used to characterize alpha granule secretion, and dense granule release were assessed using the mepacrine test.

##### Alpha Granule Secretion (CD62P/P-Selectin)

Alpha granules secretion was characterized using phycoerythrin (PE)-conjugated CD62P antibodies binding. Antibodies were added to washed platelets (2 × 10^7^ cell/mL final concentration), and cells were incubated at 37 °C for 30 min. Then, hematin (5 μM, or 30 μM) or Collagen Related Peptide (CRP, 0.5 µg/mL, 5 min as a positive control) were added to the samples and incubated at 37 °C for an additional 2 min. Finally, the reaction was stopped by the PBS (1:100), and the MFI was registered at the FL3 channel.

##### Dense Granule Secretion

To characterize the dense granule release, the widely used mepacrine test was used according to [31]. Washed platelets (2 × 10^7^ cell/mL final concentration) were loaded with FITC-conjugated mepacrine (2.5 μM, 10 min, RT), then the agonists were added (hematin 5 or 30 μM, 6 min, RT; or CRP (0.5 µg/mL, 5 min as a positive control) and finally platelets were fixed with formaldehyde (0.5% final concentration, 20 min). The reaction was stopped by PBS (1:100), and mepacrine MFI was registered at the FL1 channel. To estimate the dense granules release triggered by the agonists, the MFI in the presence of the agonists was subtracted from the MFI of the control resting platelets.

#### 2.7.3. Analysis of Phosphatidylserine Surface Exposure

Phosphatidylserine (PS) exposure on the outer platelet membrane was analyzed using Annexin-V-PE. Platelets (2 × 10^7^ cell/mL final concentration) were incubated either with hematin (5 μM, 30 μM, 10 min) or with CRP (0.5 µg/mL) together with thrombin (0.05 U/mL, 5 min) as positive controls. Subsequently, Annexin-V-PE was added to the samples (1:10), and the cell suspension was immediately diluted with Annexin-V-binding buffer (140 mM NaCl, 10 mM HEPES, 2.5 mM CaCl_2_). The samples were incubated for 10 min at RT in the dark, and then the reaction was stopped by PBS (1:100). The Annexin-V MFI was registered at the FL3 channel.

#### 2.7.4. Analysis of Platelet Viability

Calcein-AM (C-AM) was used as a marker of platelet viability based on the cell esterase activity. Platelets (2 × 10^7^ cell/mL final concentration) were incubated with hematin (5 μM, 30 μM, 20 min, RT) and then C-AM was added to cells (0.2 μM, 10 min, 37 °C). The reaction was stopped by PBS (1:40), and Calcein MFI was registered at the FL1 channel.

### 2.8. Confocal Microscopy

C-AM was additionally used for the cells’ vitality control by confocal microscopy (Leica TCS SPII, Leica Microsystems Inc., Bannockburn, IL, USA) with 63× immersion objectives. Washed platelets were stained similarly to a flow cytometric assay. Washed platelets loaded with C-AM (2 × 10^7^ cell/mL final concentration) were suspended in HEPES buffer (1.5 mM CaCl_2_) and placed in the plastic dish with further analysis of Calcein MFI at the FITC channel. Next, hematin at indicated concentrations was added to the cell suspension in the dish, and the Calcein MFI was registered. The figures were additionally zoomed in using the software to visualize the single platelets better.

### 2.9. Data Analysis

Flow cytometry data were analyzed using the original software CytExpert v2.4 (BeckmanCoulter, Brea, CA, USA). Confocal microscopy data were analyzed using the Leica TCS SPII confocal software (Leica LAS AF v.2.6, Leica Microsystems Heidelberg GmbH, Heidelberg, Germany). Data obtained by the laser diffraction method were analyzed using the original software LaSca_32 v.1498 (BioMedSystems Ltd., Saint Petersburg, Russia) of the laser particle analyzer LaSca-TM. Statistical analysis was performed in GraphPad Prism v.9 (GraphPad Software Inc., San Diego, CA, USA). The data sets were tested for normality using the Kolmogorov–Smirnov normality test. The differences between the two groups were compared either using Student’s *t*-test or the Mann–Whitney U-test. For multiple comparisons, either one-way ANOVA followed by Dunnett’s post hoc or Kruskal–Wallis test were used. Data are presented as Means ± SD. Each set of experiments was performed at least four times (*n* = 4) using at least four biological replicates, and *p* < 0.05 was considered statistically significant.

## 3. Results

### 3.1. Hematin-Induced Platelet Activation Is More Pronounced than That of Hemin

Previously we showed that hematin induced a more rapid erythrocyte spherization and hemolysis compared to hemin, indicating that hematin is a more toxic metabolite [27]. However, it remains unclear whether the effects of hematin and hemin on human platelets exhibit a similar pattern to that observed in erythrocytes. Therefore, we evaluated the effects of hematin and hemin on human platelets using the original LaSca-TM laser particle analyzer.

**Figure 1:**
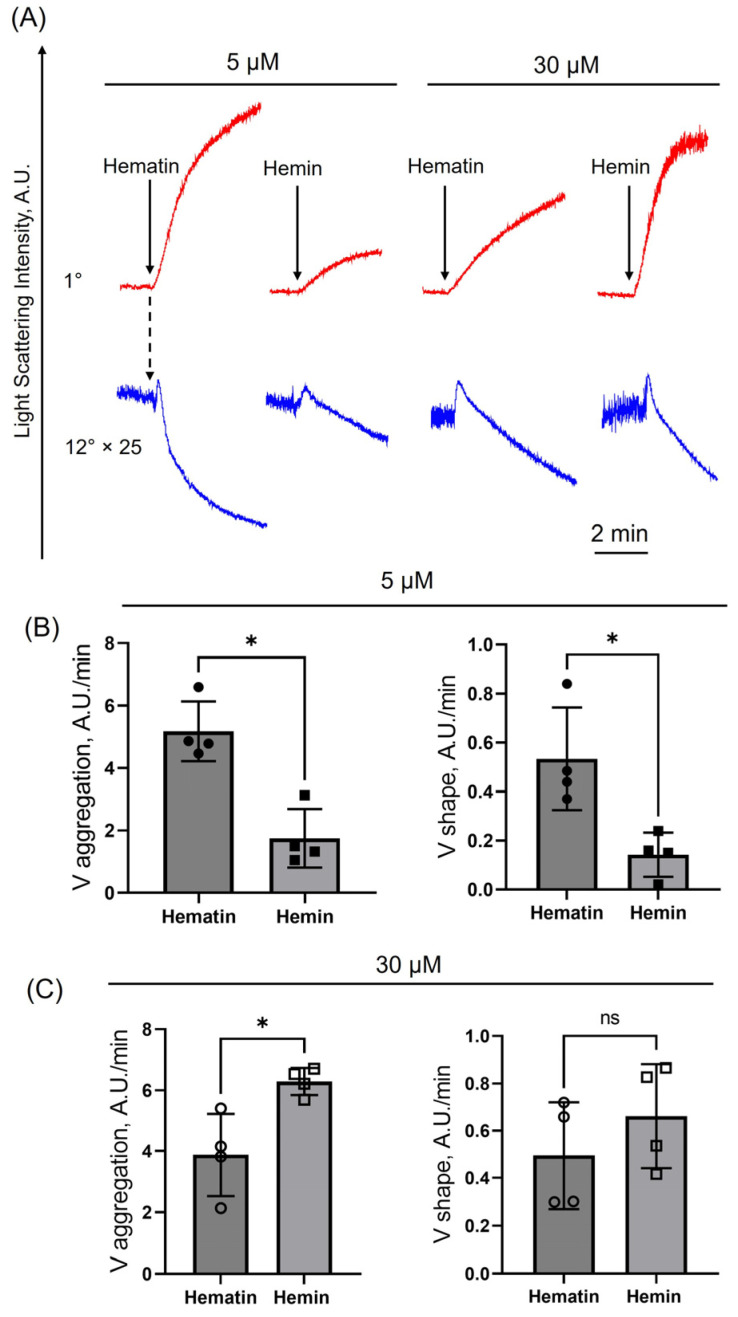
Hematin is a more potent agonist of platelet activation and aggregation compared to hemin at close to physiological concentrations in the bloodstream. Washed platelets were suspended in HEPES buffer (with 1.5 mM Ca^2+^) in the cuvette with continuous stirring (1200 rpm) at 37°. Hematin and hemin were added (black arrows) to the cells (2 × 10^7^ cells/mL) at indicated concentrations, and platelet response (shape change—initial increase in LSI at 12°, blue curve; aggregation—red curve at 1°) was registered according to scattering light intensity changes. (**A**) Representative curves of one out of five experiments using the LaSca-TM analyzer. The light scattering curves of platelet shape change (12°) were multiplied 25-fold for better visualization. (**B**) Platelet aggregation and shape change rates in the presence of 5 μM hematin or hemin. (**C**) Platelet aggregation and shape change rates in the presence of 30 μM hematin or hemin. Mann–Whitney U-test, *n* = 4, *, *p* < 0.05, ns—not significant.

At low doses (5 µM), hematin induced significantly higher velocity of a shape change and followed aggregation compared to hemin (Figure 1A,B). At high doses (30 μM), no significant differences were observed in the shape change velocity triggered by both agonists (Figure 1A,C). Hematin concentration-dependently decreased platelet transformations (shape change and aggregation velocities, Supplementary Figure S3), which is consistent with data shown in [19] and could be explained by the toxic effects of high doses of hematin in platelets (see below). Our data indicate that hematin is a more potent agonist of platelet activation and aggregation compared to hemin at close to physiological concentrations in the bloodstream [18,27]. Therefore, only the effects of hematin on platelet transformations were considered for further analysis.

### 3.2. Hematin at Low Concentrations Induces Reversible Intracellular Calcium Increase

Previously, we showed that hemin and hematin both triggered a pronounced intracellular calcium increase in human red blood cells (RBCs), which were already at nanomolar concentrations [27]. For platelets, recently, it was demonstrated that hemin (from 3 up to 50 μM) promoted a strong intracellular calcium increase, serving as an indicator for platelet activation [17,20]. However, the effects of hematin on intracellular calcium kinetics have not been elucidated.

**Figure 2:**
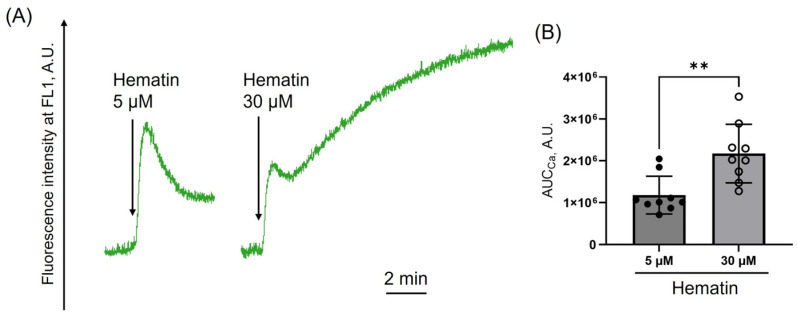
Hematin at low doses induces a reversible increase in intracellular calcium. PRP was incubated with Fluo-3 (10 μM, RT) and washed in HEPES buffer. Fluo-3-stained cells (2 × 10^7^ cells/mL) were suspended in HEPES buffer (1.5 mM Ca^2+^) in the cuvette with continuous stirring (1200 rpm) at 37°. Fluo-3 fluorescence intensity was registered at FL1 (527 nm) to visualize changes in [Ca^2+^]_i_. After registering the basal signal (2 min), hematin was added to platelet suspension at indicated concentrations (black arrows). The area under the curve (AUC_Ca_) was calculated to characterize calcium changes. (**A**) Representative curves from one of six experiments using the LaSca laser analyzer. (**B**) Quantification of the data presented in (**A**). Mann–Whitney U-test, *n* = 9, **, *p* < 0.01.

Hematin (5 μM) induced a strong rise in the intracellular calcium ([Ca^2+^]_i_) level, which decreased over time, indicating possible calcium efflux (Figure 2A). In contrast, hematin at high doses triggered a persistent increase in [Ca^2+^]_i_, demonstrating the accumulation of intracellular calcium and most likely altered efflux from the cytosol (Figure 2A,B). Thus, elevated levels of [Ca^2+^]_i_ induced by hematin at high concentrations may result in cell death due to pronounced calcium dysregulation.

### 3.3. Platelet Aggregation Induced by Low Doses of Hematin Is Inhibited by cAMP/PKA and cGMP/PKG Signaling Pathways Activation

Prostacyclin and nitric oxide (NO) represent the major platelet inhibitory signaling molecules, which promote the platelet quiescence state in the absence of blood vessel damage. Their inhibitory effects are mediated by increasing the intracellular cyclic nucleotide levels, cAMP, and cGMP, respectively. Elevated levels of cAMP and cGMP activate the corresponding protein kinases, protein kinase A (PKA) and protein kinase G (PKG), which phosphorylate multiple substrates responsible for platelet inhibition [32,33]. Recently, it was reported that activation of soluble guanylate cyclase (sGC) by riociguat inhibits platelet activation, degranulation, and aggregation induced by hemin at low doses but does not entirely reduce Ca^2+^ burst initiated by hemin at high concentrations [34]. However, it remains unknown whether hematin-induced aggregation would be inhibited by the cAMP/PKA pathway (iloprost) and whether it would be suppressed by activation of sGC (SNP) at high hematin concentrations.

**Figure 3:**
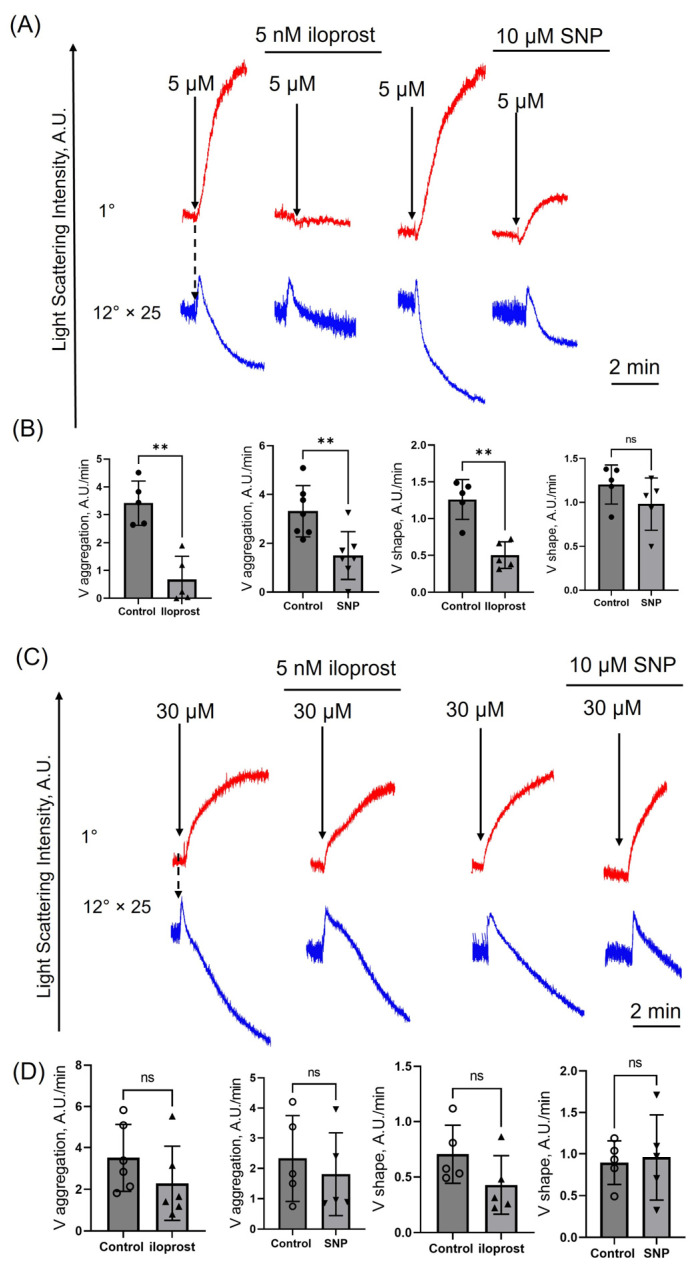
Iloprost and SNP inhibit platelet aggregation induced only by low doses of hematin. Washed platelets were suspended in HEPES buffer (1.5 mM Ca^2+^) in the cuvette with continuous stirring (1200 rpm) at 37°. Hematin (5 μM or 30 μM) was added (black arrows) to platelet suspension (2 × 10^7^ cells/mL), and aggregation (1°, red curve) and shape change (12°, blue curve) were registered as responses of control cells. Next, platelets were incubated with iloprost (5 nM) or SNP (10 μM) for 5 min, and then hematin was added (black arrows) to the cell suspension. V*_shape_* and V*_aggregation_* were calculated for each group. (**A**) Representative curves of hematin-triggered (5 µM) platelet shape change and aggregation in the absence and presence of iloprost and SNP, one out of six experiments using the LaSca laser analyzer. The light scattering curves of platelet shape change (12°) were multiplied 25-fold for better visualization. (**B**) Quantification of the data presented in (**A**). (**C**) Representative curves of hematin-triggered (30 µM) platelet shape change and aggregation in the absence and presence of iloprost and SNP, one out of six experiments using the LaSca laser analyzer. (**D**) Quantification of the data presented in (**C**). *n* = 5 ((**B**), first), *n* = 7 ((**B**), second), *n* = 5 ((**B**), third), *n* = 5 ((**B**), fourth); *n* =6 ((**D**), first), *n* = 5 ((**D**), others) Mann–Whitney U-test, *n* = 6, **, *p* < 0.01, ns—not significant.

Both iloprost and SNP inhibited platelet shape change and aggregation at low hematin doses (5 μM) (Figure 3A,B). However, platelet transformations induced by hematin at high concentrations were not suppressed by activation of PKA/PKG (Figure 3C,D), indicating that cAMP/PKA and cGMP/PKG pathways are ineffective in suppressing platelet aggregation induced by hematin at high doses.

### 3.4. Activation of PKA/PKG Inhibits Calcium Increase Induced Only by Low Doses of Hematin

Next, we checked whether activation of cAMP/cGMP signaling pathways would inhibit hematin-induced [Ca^2+^]_i_ rise in platelets.

**Figure 4:**
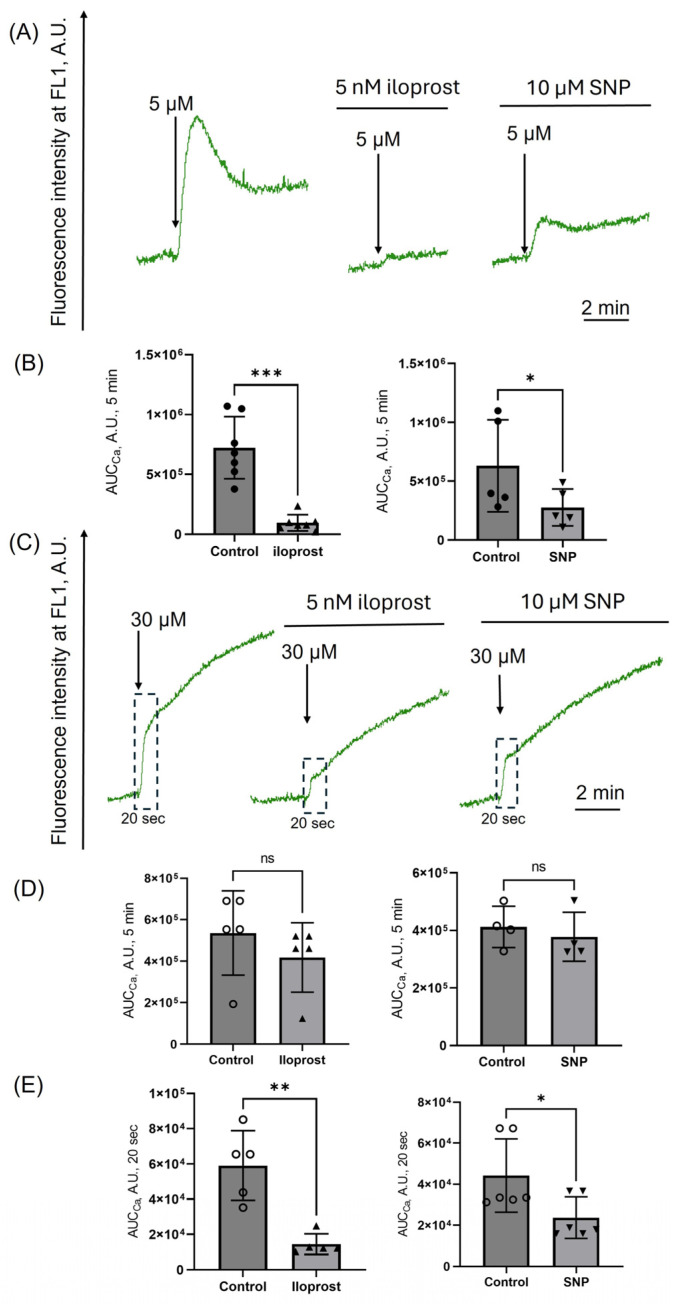
Iloprost and SNP inhibit calcium increase induced only by low doses of hematin. PRP was incubated with Fluo-3 (10 μM, RT) and then washed once in HEPES buffer. Fluo-3-stained cells (2 × 10^7^ cells/mL) were suspended in HEPES buffer (1.5 mM Ca^2+^) in the cuvette with continuous stirring (1200 rpm) at 37°. Fluo-3 fluorescence intensity was registered at FL1 to visualize the changes in [Ca^2+^]_i_. After the registration of the basal signal (2 min), hematin (black arrows) was added to platelet suspension at indicated concentrations to characterize the intracellular calcium response of control cells. Next, platelets were incubated with iloprost (5 nM) or SNP (10 μM) for 5 min, and then hematin was added to the cell suspension. The sustained AUC*_Ca_* (5 min) (**B**,**D**) and initial AUC*_Ca_* (20 s) responses (**E**) were calculated to quantitatively characterize the effects of iloprost and SNP on hematin-induced platelet [Ca^2+^]_i_ changes. (**A**,**C**) Representative curves of hematin-triggered calcium changes in the absence and presence of iloprost and SNP, one out of six experiments using the LaSca laser analyzer. (**B**) Quantification of the data presented in (**A**). (**D**,**E**) Quantification of the data presented in (**C**). Mann–Whitney U-test, *n* = 7 ((**B**), first), *n* = 5 ((**B**), second); *n* =5 ((**D**), first), *n* = 4 ((**D**), second); *n* = 5 ((**E**), first), *n* = 6 ((**E**), second), * *p* < 0.05, **, *p* < 0.01, ***, *p* < 0.001, ns—not significant.

Iloprost almost completely and SNP partially inhibited [Ca^2+^]_i_ increase at low hematin doses (Figure 4A,B), which directly correlates with the inhibition of platelet aggregation (Figure 3A,B). However, the effects of iloprost and SNP at high hematin concentrations (Figure 4C) were more complex. To properly characterize it, we analyzed two parameters: (a) AUC*_Ca_* during 5 min (Figure 4D), which describes the sustained [Ca^2+^]_i_ increase response, and (b) AUC*_Ca_* during the first 20 s, which corresponds to the initial [Ca^2+^]_i_ increase response (Figure 4D). Iloprost and SNP significantly inhibited the initial rise of [Ca^2+^]_i_ without preventing the sustained [Ca^2+^]_i_ increase. These data indicate that preventing initial [Ca^2+^]_i_ increase is insufficient for inhibiting a high-dose hematin-induced platelet aggregation by activating PKA/PKG pathways (Figure 3C,D).

### 3.5. Hematin at Both Low and High Doses Induces Platelet Aggregation but Not Agglutination

**Figure 5:**
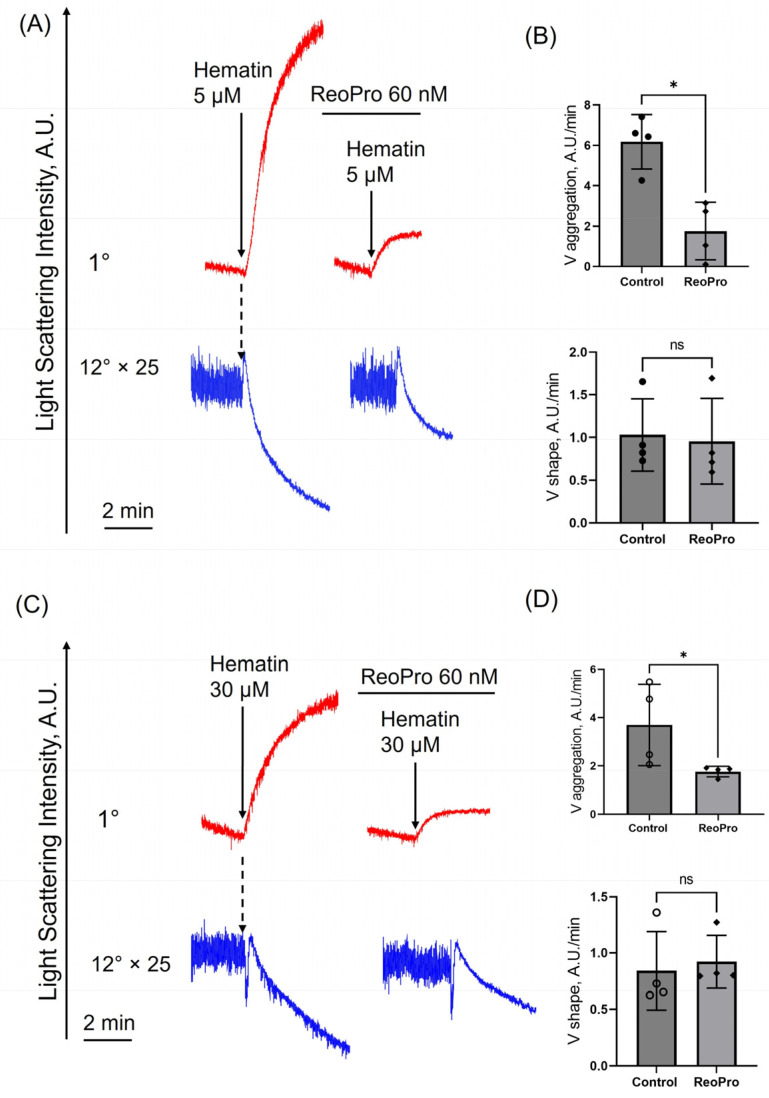
Hematin induces platelet shape change and aggregation but not agglutination. Washed platelets were suspended in HEPES buffer (1.5 mM Ca^2+^) in the cuvette with continuous stirring (1200 rpm) at 37°. Hematin (5 μM or 30 μM) was added (black arrows) to platelet suspension (2 × 10^7^ cells/mL), and aggregation (1°, red curve) and shape change (12°, blue curve) corresponding to the control platelet response were registered. Next, platelets were incubated with ReoPro (60 nM, 5 min), and then hematin was added to the cell suspension. V*_aggregation_* and V*_shape_* were calculated to characterize platelet transformations quantitatively. (**A**) Representative curves of hematin-triggered (5 μM) platelet transformations in the absence and presence of ReoPro, one of six experiments using the LaSca laser analyzer. The light scattering curves of platelet shape change (12°) were multiplied 25-fold for better visualization. (**B**) Quantification of the data presented in (**A**). (**C**) Representative curves of hematin-triggered (30 μM) platelet transformations in the absence and presence of ReoPro, one of six experiments using the LaSca laser analyzer. (**D**) Quantification of the data presented in (**C**). Mann–Whitney U-test, *n* = 4, *, *p* < 0.05, ns—not significant.

Previously, it was shown that hemin at low concentrations triggers platelet aggregation, whereas at high concentrations, it potentially induces platelet agglutination [16]. Platelet aggregation and agglutination fundamentally differ in their mechanisms. Aggregation requires the activation of integrin αIIbβ3, followed by fibrinogen and platelet interactions, whereas agglutination results from passive cross-linking of adjacent platelets via GPIbα [35]. To determine whether hematin at high concentrations causes agglutination, we used αIIbβ3 inhibitor ReoPro. ReoPro suppressed platelet aggregation at both low and high hematin concentrations but did not affect platelet shape change (Figure 5A–D). The platelet response to ADP (used as a positive control) in the presence of αIIbβ3 integrin inhibitor was identical, and the shape change remained unchanged, as expected (Supplementary Figure S4). However, ReoPro completely abolished ADP-induced platelet aggregation but incompletely inhibited the platelet aggregation in the presence of hematin, indicating the possible, however, not significant agglutination of platelets. Thus, low and high hematin concentrations mostly triggered platelet aggregation but not agglutination.

### 3.6. Hematin Decreases Platelet Viability Only at High Concentrations

Previously, oxidized heme derivatives were shown to intercalate into the cell membrane and induce reactive oxygen species generation, followed by lipid peroxidation through the Fenton reaction [19,36]. Hemin induced ferroptosis in human platelets at high concentrations, indicating these compounds’ toxic properties [19]. However, the effects of hematin on human platelet viability at low concentrations have not been elucidated yet.

**Figure 6:**
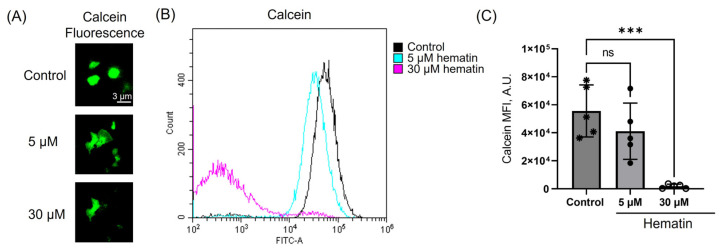
Hematin decreases platelet viability only at high concentrations. Washed platelets (2 × 10^7^ cells/mL) were incubated with indicated concentrations of hematin, then stained by C-AM (0.25 μM, 10 min, 37 °C), and calcein fluorescence was analyzed by confocal microscopy at FITC channel (**A**) and flow cytometry (**B**). (**C**) Quantification of the data presented in (**B**). One-way ANOVA, Dunnet’s test, *n* = 5, ***, *p* < 0.001, ns—not significant. Original dot plots are presented on the Supplementary Figure S7.

Hematin at low doses (5 μM) induced only platelet transformations (Figure 1, Figure 3 and Figure 5), whereas platelet intracellular esterase activity, or vitality, was not affected (Figure 6B,C). In contrast, hematin at high doses (30 μM) significantly reduced calcein fluorescence (Figure 6A), indicating a strong decline in platelet viability. Thus, low hematin concentrations are involved only in platelet activation and aggregation; however, high doses of hematin exhibit strong cytotoxic effects on cells.

### 3.7. Hematin Triggers Phosphatidylserine Exposure Only at High Concentrations

Potent agonists, such as thrombin or collagen, induce externalization of phosphatidylserine, an established marker of cell death, on the platelet surface, leading to enhanced procoagulant activity, which is crucial for thrombin generation and hemostasis [37]. Previously, it was shown that hemin induced PS exposure in a dose-dependent manner, and only high concentrations induced membrane reorganization [21]. However, the effects of hematin on platelet PS exposure remained unknown.

**Figure 7:**
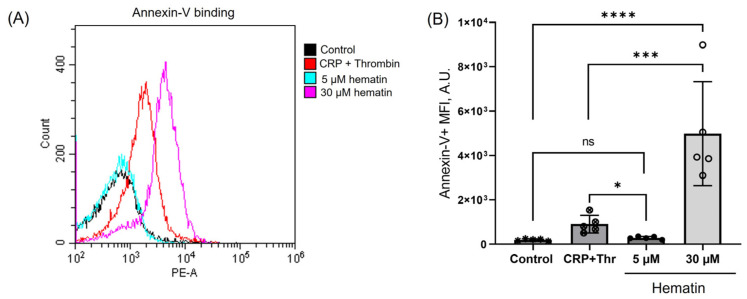
Hematin triggered a significant PS exposure only at high concentrations. Washed platelets (2 × 10^7^ cells/mL) were incubated with indicated concentrations of hematin, and then stained by Annexin V-PE (0.25 μM, 10 min, 37 °C) and analyzed by flow cytometry to register the response of the control cells. Additionally, as a positive control of PS exposure, platelets were treated with CRP in combination with thrombin (0.5 µg/mL, 0.05 U/mL, 5 min) with the following staining with Annexin-V and analysis by flow cytometry. (**A**). Quantification of the data presented in (**B**). One-way ANOVA, Dunnett’s test, *n* = 5, * *p* < 0.05, ***, *p* < 0.001, ****, *p* < 0.0001. Original dot plots are presented on the Supplementary Figure S7.

Hematin at low concentrations (5 μM) did not increase Annexin-V fluorescence compared to control, indicating no alteration in phospholipid asymmetry (Figure 7A). In contrast, hematin at high concentrations (30 μM) induced a significant increase in Annexin-V fluorescence, i.e., release of PS to the platelet surface and formation of the procoagulant platelets. Interestingly, hematin at high concentrations triggered a more pronounced externalization of PS compared to simultaneous CRP and thrombin supplementation (Figure 7B). These findings are consistent with the previously described results demonstrating a significant decrease in platelet viability (see Section 3.6) and indicate the same pattern of cytotoxic effects shown for hemin by others [20].

### 3.8. Hematin Induces Dense but Not Alpha Granule Secretion

Upon activation, platelets secrete active substances from their intracellular granules. Dense granules contain low-molecular-weight compounds (e.g., ADP, serotonin, and calcium) which potentiate platelet activation, while α-granules concentrate substances like P-selectin, von Willebrand factor (vWF), fibrinogen, coagulation factors, etc. [38]. Previously, it was demonstrated that hemin at low concentrations induces dense granules secretion, while at high concentrations—P-selectin shedding [39,40]. However, whether hematin stimulates either alpha or dense granule secretion has not been addressed yet.

**Figure 8:**
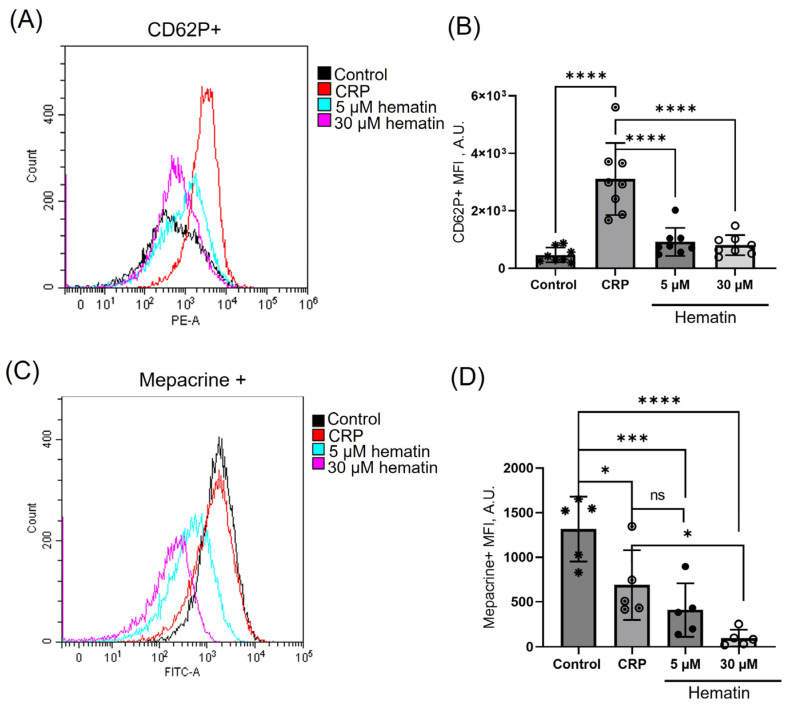
Hematin induces dense but not alpha granule secretion. Washed platelets (2 × 10^7^ cells/mL) were incubated with CD62P (1:20, 30 min, 37 °C) or stained by mepacrine (2.5 µM, 10 min, RT). Then, hematin (5 μM or 30 μM) or CRP (0.5 µg/mL, 2 min, 37 °C) were added to cells and analyzed by flow cytometry. In the mepacrine assay, platelets were also fixed with formaldehyde (0.5%). Representative histograms of CD62P+ cells distribution by count and MFI and intracellular mepacrine accumulation registered by flow cytometry are shown (**A**,**C**) with corresponding quantitative analysis (**B**,**D**). One-way ANOVA, Dunnett’s test, *n* = 8 (**B**), *n* = 5 (**D**), *, *p* < 0.05, ***, *p* < 0.001, ****, *p* < 0.0001, ns—not significant. Original dot plots are presented in Supplementary Figure S7.

CRP, as expected, induced a prominent increase in CD62P/P-selectin fluorescence, indicating a significant increase in alpha-granule secretion. Hematin, however, led to a slight but not significant MFI increase at both low and high concentrations (Figure 8A,B). At the same time, the secretion of dense granules was dramatically elevated at both low and high concentrations of hematin. The observed effect was even more pronounced than dense granule release upon CRP stimulation (Figure 8C,D). These data demonstrate that hematin effects on dense granule secretion are similar to that of hemin. However, the question of alpha granule secretion merits future examinations regarding P-selectin shedding from the platelet surface.

### 3.9. Albumin Moderates Hematin-Induced Platelet Activation

Albumin, being the major protein component of blood plasma, was reported to bind ferric heme derivatives at a quantification ratio of 1:1, suppressing their peroxidative and catalytic effects [41]. Previously, we and others showed that albumin counteracted hemin/hematin effects on RBCs: (a) erythrocytes returned to discoid form from the spheric state, and (b) hemin trapped in the erythrocyte membrane was extracted upon albumin supplementation [42,43]. However, whether albumin would have a similar effect on human platelet activation and aggregation remains unknown.

**Figure 9:**
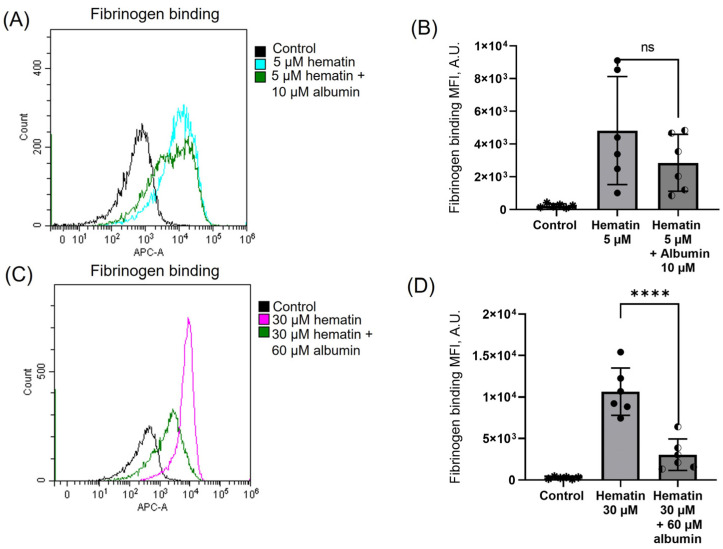
Hematin-induced platelet activation decreases in the presence of albumin. Washed platelets (2 × 10^7^ cells/mL) were incubated with Fibrinogen-APC (15 µg/mL, 30 min, 37 °C), then hematin (5 or 30 μM) was added to the cells and analyzed by flow cytometry. Albumin (10 or 60 μM) was added to stained cells after incubation (5 min) with hematin. Shown are representative data by flow cytometry (**A**,**C**) and quantitative analysis (**B**,**D**). Mann–Whitney U-test, *n* = 6, ****, *p* < 0.0001, ns—not significant. Original dot plots are presented in Supplementary Figure S7.

Albumin (10 μM) addition to platelets treated with hematin (5 min) slightly decreased fibrinogen binding, i.e., platelet activation induced by hematin at low concentrations (Figure 9A,B). At the same time, albumin supplementation (60 μM, 5 min after hematin) considerably attenuated platelet activation triggered by hematin at high doses (Figure 9C,D). These data indicate that, in contrast to RBCs, where albumin addition prevented or reversed hematin-induced transformation [27], in platelets, it only moderately (especially at low doses of hematin) reduced platelet activation.

### 3.10. Albumin Induces Disaggregation in Hematin-Treated Platelets

**Figure 10:**
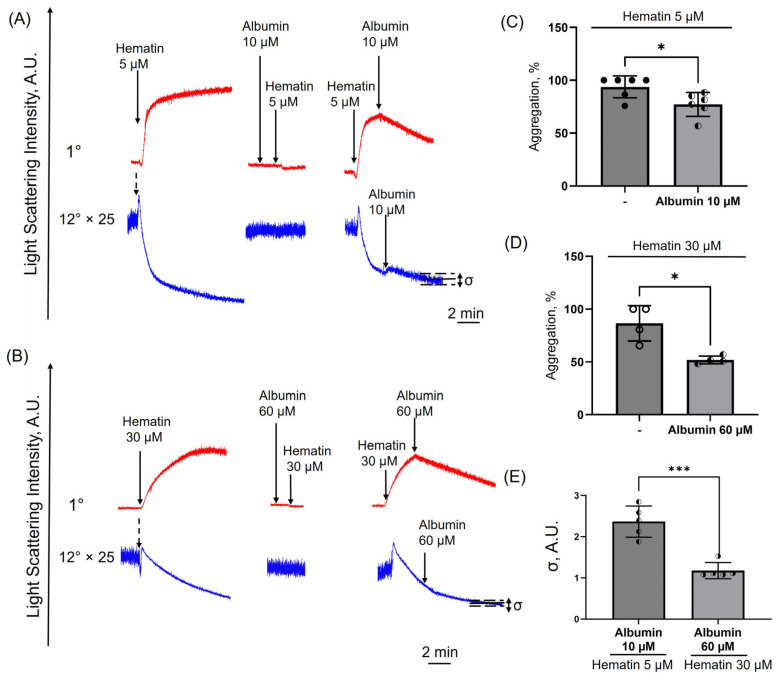
Albumin supplementation initiated platelet disaggregation processes. Washed platelets (2 × 10^7^ cells/mL) were suspended in HEPES buffer (1.5 mM Ca^2+^) in the cuvette with continuous stirring (1200 rpm) at 37°. Hematin in low (**A**) and high (**B**) concentrations was added to the cells (black arrows), and aggregation (1°, red curve) and shape change (12°, blue curve) corresponding to the control platelet response were registered. Albumin in 2-fold higher concentration than hematin, 10 and 60 μM correspondingly, was added upon reaching the maximum aggregation signal. Shown are representative curves of platelet transformation registered by laser diffraction.The light scattering curves of platelet shape change (12°) were multiplied 25-fold for better visualization. (**A**,**B**). Oscillations width (δ) was calculated on hematin alone and hematin + albumin samples in (**A**,**B**). (**C**,**D**) the corresponding quantitative analysis of the % of aggregation and (**E**) δ AU. Mann–Whitney U-test, *n* = 6 (**C**), *n* = 4 (**D**), *n* = 5 (**E**), *, *p* < 0.05, ***, *p* < 0.001.

Next, we asked whether albumin could induce disaggregation in hematin-treated platelets. Albumin added prior to hematin (2 min) completely prevented platelet activation for both low and high hematin concentrations (Figure 10A,B). To evaluate the effects of albumin on the disaggregation processes, the albumin was added when hematin-induced platelet aggregation reached maximum values (maximum LSI corresponding to 100% aggregation). Albumin addition led to a significant decrease in the LSI signal at low and high hematin concentrations, indicating the initiation of platelet disaggregation (Figure 10C,D).

Previously, using the laser diffraction and flow cytometry methods [27], we showed that the LSI oscillation width (δ) could be used to describe morphological changes in human erythrocytes. Therefore, we assumed that the changes in the LSI oscillation width at 12° similarly characterize changes in platelet shape. Albumin supplementation restored platelet shape changed by hematin at low concentrations, whereas it did not prevent changes induced by high doses of hematin (Figure 10E). The addition of albumin to the platelets treated with 5 µM of hematin probably moderated platelet transformation or stabilized already transformed cells. In contrast, albumin supplementation did not reverse platelet transformation induced by high hematin concentrations. These data indicate that as well as in the case with human erythrocytes, the effects of low-dose hematin on platelets could be partially reversed in the presence of albumin, but high hematin concentrations led to irreversible platelet transformation.

## 4. Discussion

Recently, free heme derivatives, such as hemin, etc., have been identified as potent factors triggering platelet activation and aggregation, thus leading to thrombus formation or cell elimination by macrophages [18]. However, considering the etiology of the pathology (hemolytic disorders, parasite invasion, gastric problems, or porphyria treatment), the plasma scavenging system (haptoglobin, hemopexin, and albumin) would be challenged either by the overwhelming formation of hemin and hematin [44]. Therefore, here first, we assessed whether the effects of hemin and hematin on human platelets would be similar or whether structural and physicochemical differences would lead to specific responses. We showed that both compounds trigger strong activation and aggregation of platelets, however, hematin administration triggered a more pronounced effect on the platelet transformations in sub-physiological doses (Figure 1). Platelet activation induced by high hematin doses was less pronounced than low doses (Supplementary Figure S3), but this was not accompanied by the platelet count reduction (CD41+ staining, Supplementary Figure S5) [30].

The tight balance between activation and inhibition is crucial to maintain proper platelet function. Previous studies have shown hemin to be a ligand of CLEC-2 and GPVI platelet receptors, which acts through the SFK-SYK-PLCγ2 signaling pathway [15,16,37]. The CLEC-2 signaling pathway is associated with secondary mediators (ADP, Thromboxane A2), but cyclooxygenase or P2Y_12_ inhibitors did not suppress hemin-induced platelet activation in contrast to CLEC-2 agonist podoplanin [16]. We also confirmed this for hematin by showing that P2Y_12_ (ARC) and thromboxane A2 (SQ29548) receptor antagonists do not affect hematin-induced aggregation (Supplementary Figure S6), indicating the involvement of other receptors in hematin-induced platelet activation. For instance, glycoprotein Ibα (GPIbα), the ligand-binding subunit of platelet GPIb-IX complex, interacting with von Willebrand factor was also shown to bind hemin and regulate platelet clearance during hemolytic disease [18].

Intracellular calcium signaling activation is essential for platelet transformations, including shape change, degranulation, and aggregation [18,21]. Typically, intracellular calcium rise is followed by rapid calcium efflux by plasma membrane and endoplasmic ATPases (plasma membrane Ca^2+^ ATPases, PMCA; Sarcoendoplasmic Reticulum Calcium ATPases, SERCA), preventing excessive activation of platelets [45]. Hemin and hematin were recently shown to induce apoptosis and necroptosis in RBCs by increasing intracellular Ca^2+^ levels, while RBCs transformation was Ca^2+^ independent [10]. Here, we showed that in human platelets, hematin (5–30 µM), acting similarly to hemin [11], provoked rapid Ca^2+^ increase (Figure 2). At low hematin concentrations, the response was reversible/balanced, while at high concentrations, no decrease was detected, only a persistent intracellular calcium accumulation in the cytosol, indicating the impairment of the calcium efflux system. The detected intracellular calcium dysregulation could participate in the cytotoxic effects of hematin and explain impaired platelet functions, leading to thrombosis and thrombocytopenia.

cAMP/PKA and cGMP/PKG pathways are two major systems responsible for platelet inhibition [32,33]. Elevated levels of cGMP (DEA/NO-riociguat) were shown to decrease intracellular Ca^2+^ release, platelet degranulation (P-selectin), and ex vivo thrombus formation upon hemin stimulation [34]. We showed that cGMP and cAMP activation (iloprost and SNP, respectively) inhibited platelet aggregation only at low doses of hematin in contrast to high hematin concentrations, where these inhibitors were ineffective (Figure 3). Using the original laser diffraction method, the induced by hematin changes of [Ca^2+^]_i_ in platelets were analyzed more thoroughly. We showed, for the first time, that PKA/PKG activation inhibited initial hematin-induced [Ca^2+^]_i_ rise (within 20 s), but the sustained calcium increase (5 min) remained unchanged, indicating the irreversible platelet transformation in the presence of high hematin doses.

High doses of hemin were previously described to cause ferroptosis, a non-apoptotic platelet cell death associated with increased formation of reactive oxygen species, leading to lipid peroxidation and subsequent plasma membrane rupture [20]. We showed that low hematin concentrations do not alter intracellular esterase activity (Figure 6) or provoke PS externalization (Figure 7), indicating only platelet transformations but not cell death. However, high hematin concentrations significantly reduced platelet esterase activity (vitality) and triggered a pronounced PS exposure. Notably, PS externalization was significantly higher than that induced by collagen-related peptide and thrombin, which were used as a positive control of procoagulant platelet phenotype formation. Thus, these findings go along with the previous result on calcium regulation and indicate that high hematin concentrations contribute to platelet death.

It was previously assumed that hemin at high concentrations induces platelet agglutination, possibly explaining the differences between the effects of low and high hemin concentrations [16]. However, in our experiments, inhibition of aIIbβ3 integrins significantly suppressed hematin-induced platelet aggregation at low and high hematin doses (Figure 5), indicating that hematin triggers only platelet aggregation but not agglutination.

Under normal physiological conditions, albumin participates in scavenging free ferric protoporphyrin IX in the circulation, preventing the toxic effects of heme derivatives [44]. However, in hemolytic complications, hemin/hematin concentrations could exceed the binding capacity of free heme scavengers, increasing up to 50 µM in circulation [9] and even up to 3 mM locally in the hematoma area [46], resulting in the insufficiency of the scavenging system. In our previous study, we demonstrated that albumin not only prevented hemin and hematin-induced transformation of erythrocytes but also restored their normal discoid shape [27]. Here, we showed that in the presence of albumin, hematin-induced activation was moderated, and albumin administration to aggregated platelets initiated platelet disaggregation processes (Figure 10).

## 5. Conclusions

Uncontrolled hemin/hematin-induced platelet activation and procoagulant activity represent critical factors that may elevate the risk of comorbidities such as intravascular thrombus formation, myocardial infarction, and ischemic stroke in hemolytic diseases. Although numerous aspects of hemin-induced platelet transformations have already been described, this study added additional information concerning free ferric protoporphyrin IX effects on platelets. Here, using an original analytical platform (laser diffraction-based particle analyzers LaSca-TM and -TMF) adjusted for platelet activation analysis, we showed the similarities of hemin and hematin effects on human platelet activation. Furthermore, we identified significant diversity in the effects of hemin and hematin on platelets, providing a detailed characterization of hematin’s impact on platelet activation, viability, dynamics of intracellular calcium mobilization, and its modulation by albumin. The unraveling and understanding of distinct signaling pathways underlying hemin- and hematin-induced platelet activation and transformation is especially relevant in the context of hemolytic and bacterial invasion-related diseases. These findings have potential clinical implications, especially for conditions such as malaria and porphyria, where hematin predominates. A deeper understanding of these mechanisms could improve the development of therapeutic strategies for managing platelet dysfunction and thrombotic complications in the aforementioned pathologies.

### Limitations of the Study

All experiments were conducted in either washed platelets or diluted PRP, with albumin concentrations significantly lower than those described for human plasma [47]. Thus, these experimental conditions are artificial and cannot yet be directly compared to actual physiological conditions within the circulation. Moreover, albumin is not the only player in free heme scavenging. Therefore, the effects and roles of other free heme scavenging proteins such as haptoglobin, hemopexin, and both high- and low-density lipoproteins [41,48] should be tested in future studies.

Here, we only addressed the effects of ferric protoporphyrin IX on platelet activation, however, a comprehensive analysis of the distinct mechanisms underlying the disruption of functional cell structures is crucial to develop strategies to mitigate free heme toxicity in circulating blood cells. Another problem is that currently, there are no definitive data on the in vivo ratio of hemin/hematin, nor are there any data on the particular conditions of its formation. Addressing these questions is critical for advancing our understanding of free heme dynamics in physiological and pathophysiological states.

## Data Availability

The data underlying this article will be shared at reasonable request to the corresponding author.

## Supplementary Materials

The following supporting information can be downloaded at https://www.mdpi.com/article/10.3390/cells14040255/s1, Figure S1: Graphic explanation of aggregation (A) and shape change (B) velocities determination; Figure S2: Characterization of intracellular calcium dynamics upon agonist treatment.; Figure S3: Low hematin concentrations induce more rapid shape change and aggregation than high concentrations.; Figure S4: ADP-induced platelet aggregation is completely inhibited in the presence of αIIbβ3 integrin inhibitor ReoPro.; Figure S5: Hematin administration did not affect platelet count; Figure S6: Hematin-induced platelet activation is independent of ADP and TxA2 signaling; Figure S7: Original dot plots for Flow Cytometry experiments.
