# Supplementary material for: Analysis of Ferric Protoporphyrin IX Effects on Human Platelets: Hematin Is a More Potent Agonist than Hemin
Source: Cells. 2025 Feb 11;14(4):255. doi: 10.3390/cells14040255 (PMC11853094; doi:10.3390/cells14040255)
Supplement: Supplementary file 1 [file cells-14-00255-s001.zip › cells-3423777-supplementary.pdf]

**Supplementary Materials**

## S1. Analysis of platelet aggregation and shape change by the original laser diffraction method

Previously, we thoroughly described the principles of the laser diffraction method for analysis of platelet shape change and aggregation; for the details, see the manuscript and supplementary materials in [1]. Briefly, the LaSca-TM laser analyzer registers scattered by platelet suspension light and its intensity (light scattering intensity, LSI) at the scattering angles from 1° to 12°. Based on the light scattering indicatrix, the angles 1° and 12° were chosen to describe platelet aggregation and shape change. The addition of platelet agonists induces a rapid increase of LSI signal at 12°, corresponding to the shape change of platelets (Figure S1A). The rise of LSI at 1° and simultaneous decrease at 12° corresponds to platelet aggregation (Figure S1B). The platelet disaggregation was characterized as a decline of LSI at 1° after reaching 100% Aggregation (Figure S1C).

To quantitatively characterize these processes, we calculated from the LSI curves:

- the velocity of aggregation ($V_{aggregation}$);
- the velocity of shape change ($V_{shape}$);
- the aggregation %

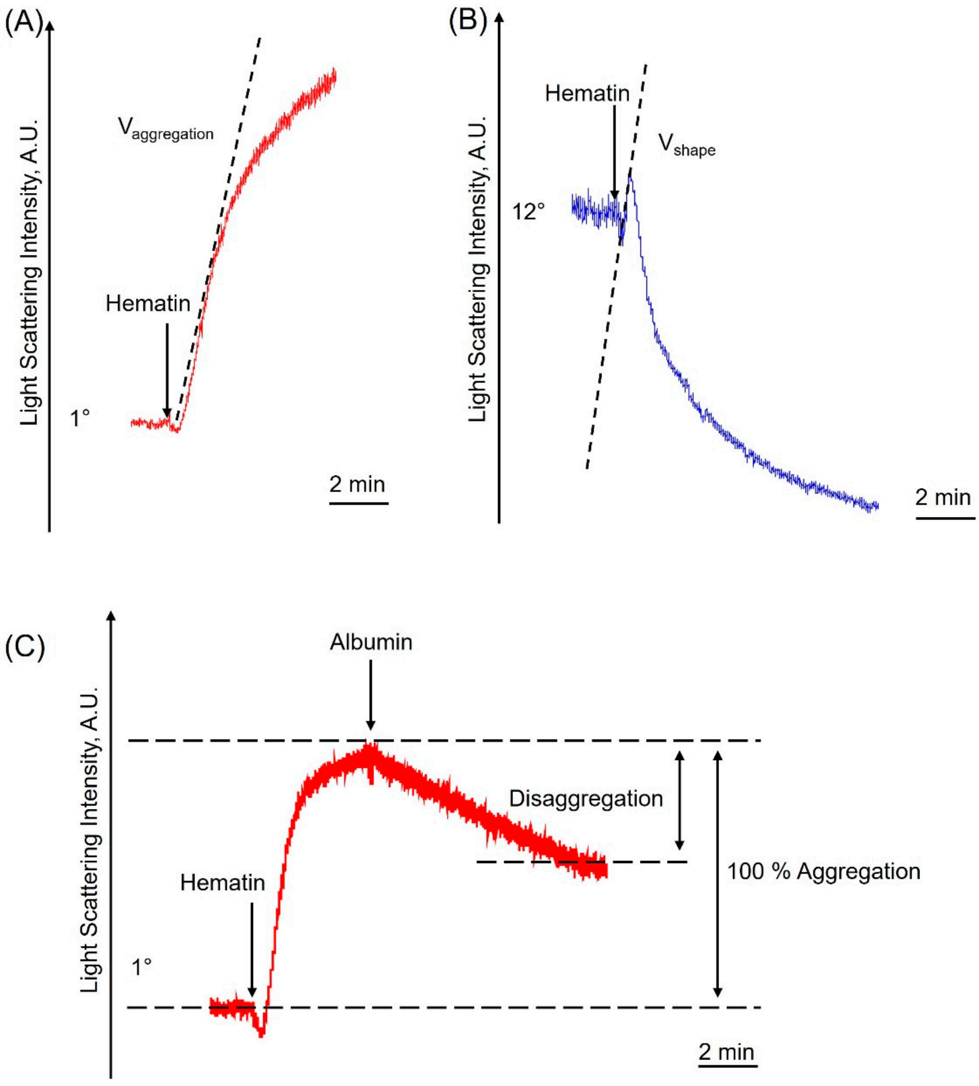

**Figure S1. Graphic explanation of aggregation (A) and shape change (B) velocities parameters and their determination.** Washed platelets were suspended in HEPES buffer (1.5 mM $Ca^{2+}$) in the cuvette with continuous stirring (1200 rpm) at 37°. Hematin (5 µM, black arrow) was added to platelet suspension (2 x $10^7$ cells/mL), and aggregation (1°) and shape change (12°) was registered according to the LSI changes. (A) Aggregation velocity ($V_{aggregation}$) and (B) shape change velocity ($V_{shape}$) were calculated in the original software LaSca_32 v.1498. (C) The 100% aggregation percent was taken as a maximum LSI signal, and albumin-triggered disaggregation was calculated as a decrease of the maximum LSI signal.

## S2. Analysis of intracellular calcium mobilization by the original laser diffraction method

To monitor the intracellular calcium changes, we used a novel laser microparticle analyzer, LaSca-TMF, with a 488 nm laser and a fluorescence detector of 527 nm (FL1). A detailed description of the method is presented in [1]. In brief, PRP was incubated with Fluo-3-AM (10 µM, 60 min, RT, in the dark) with further centrifugation (2400 RPM, 4 min), and obtained platelet pellets were resuspended in HEPES buffer (2 x $10^7$ cell/mL final concentration) and analyzed. The increase of the FL1 signal corresponded to intercellular calcium rise. To characterize intracellular calcium mobilization dynamics, we calculated the area under the curve ($AUC_{Ca}$) either for the initial (5s) or the sustained (5 min) $Ca^{2+}$ response (Figure S2).

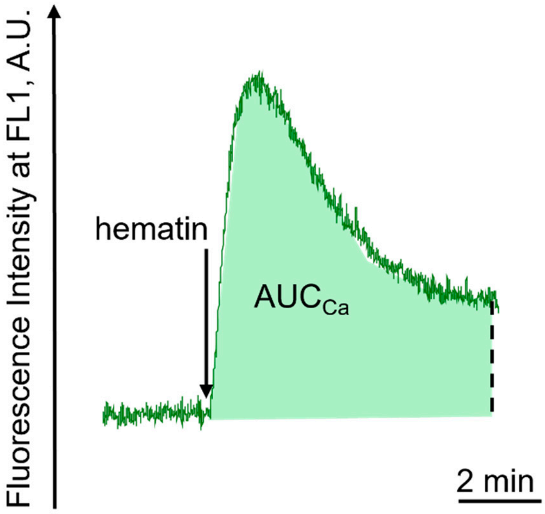


**Figure S2. Characterization of intracellular calcium dynamics upon agonist treatment.** PRP was incubated with Fluo-3-AM (10 µM, 60 min, RT, in the dark), centrifuged (2400 RPM, 4 min), and platelet pellets were resuspended in HEPES buffer. Then, washed and stained platelets (2 x $10^7$ cell/mL final concentration) were analyzed with Fluo-3 fluorescence detection. The area under the curve ($AUC_{Ca}$) was calculated using the original software LaSca_32 v.1498.

## S3. Low-dose hematin administration induced a more rapid platelet shape change and aggregation than high-dose

Previously, it was shown that hemin at low concentrations induced a more rapid platelet aggregation than at high doses [2]. We confirmed this effect for hematin and demonstrated that low hematin concentrations induce a more rapid shape change (Figure S4).

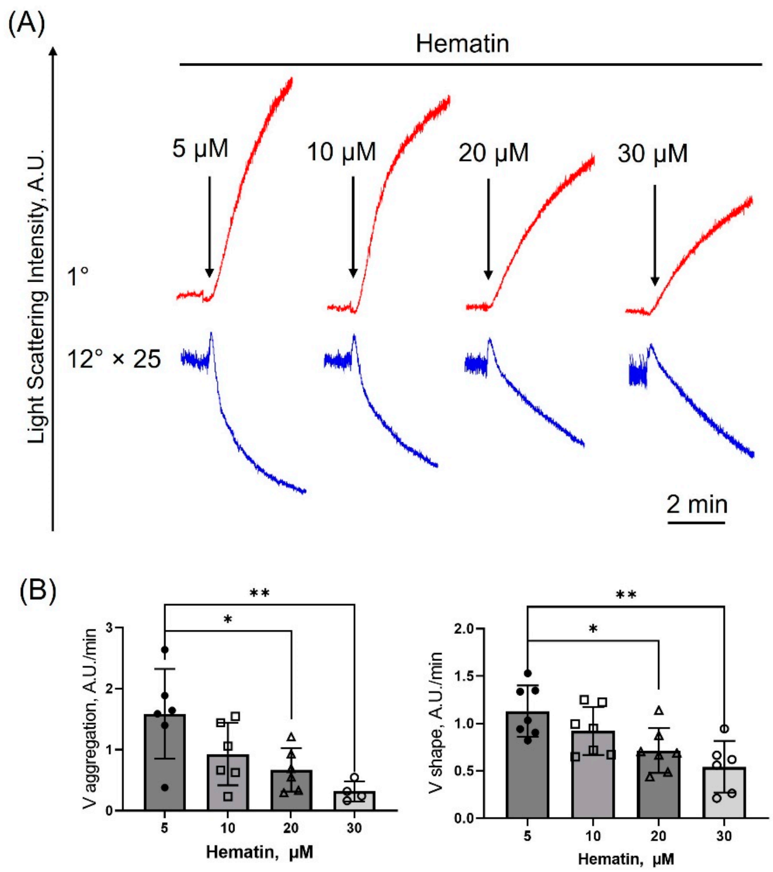


**Figure S3. Low hematin concentrations induce more rapid shape change and aggregation than high concentrations.** Washed platelets were suspended in HEPES buffer (1.5 mM $Ca^{2+}$) in the cuvette with continuous stirring (1200 rpm) at 37°. Hematin (5-30 μM) was added to platelets (black arrows), and aggregation (1°) and shape change (12°) was registered. (A) Representative curves from one of six laser diffraction experiments. (B) quantification of the data presented in (A). Velocities of platelet shape change and aggregation after administration of hematin at indicated concentrations. One-way ANOVA, Dunnet's test, *, $p<0.05$, **, $p<0.01$.

## S4. ADP-induced platelet aggregation is completely inhibited in the presence of αIIbβ3 integrin inhibitor ReoPro

The platelet response to ADP (used as a positive control) in the presence of αIIbβ3 integrin inhibitor ReoPro was identical, and the shape change remained unchanged, as expected (Figure S6).

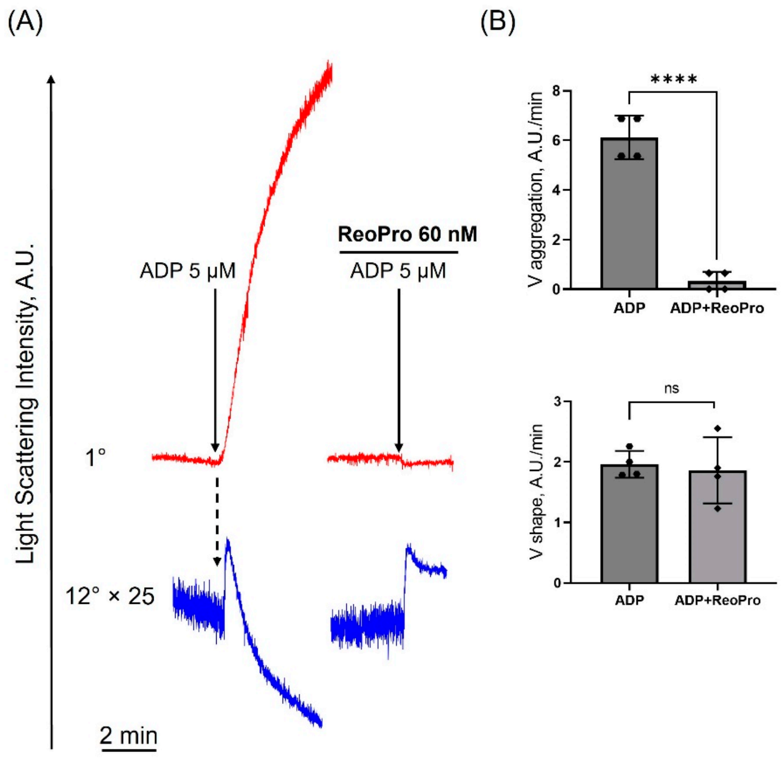


**Figure S4. ADP-induced platelet aggregation is completely inhibited in the presence of αIIbβ3 integrin inhibitor ReoPro.** PRP was suspended in HEPES buffer (1.5 mM $Ca^{2+}$) in the cuvette with continuous stirring (1200 rpm) at 37°. ADP at indicated concentrations was added to PRP or platelet suspension (2 x $10^7$ cells/mL) corresponding to control platelet response, and aggregation (1°) and shape change (12°) were registered. Next, platelets were incubated with ReoPro (60 nM, 5 min), and then ADP was added to the cell suspension. $V_{aggregation}$ and $V_{shape}$ were calculated to characterize platelet transformations quantitatively. (A) Representative curves of ADP-triggered (5 μM) platelet transformations in the absence and presence of ReoPro, one of six experiments using the LaSca laser analyzer. (B) Quantification of the data presented in (A). Mann-Whitney U-test, n=4, ****, p<0.0001, ns – not significant.

## S5. Hematin administration did not affect platelet count

CD41 is a convenient marker for the identification of platelets [3]. Platelets were gated according to the CD41 positive events (A). Hematin treatment did not alter the CD41+ event count, which indicated the unchanged cell count (Figure S2A, B, C).

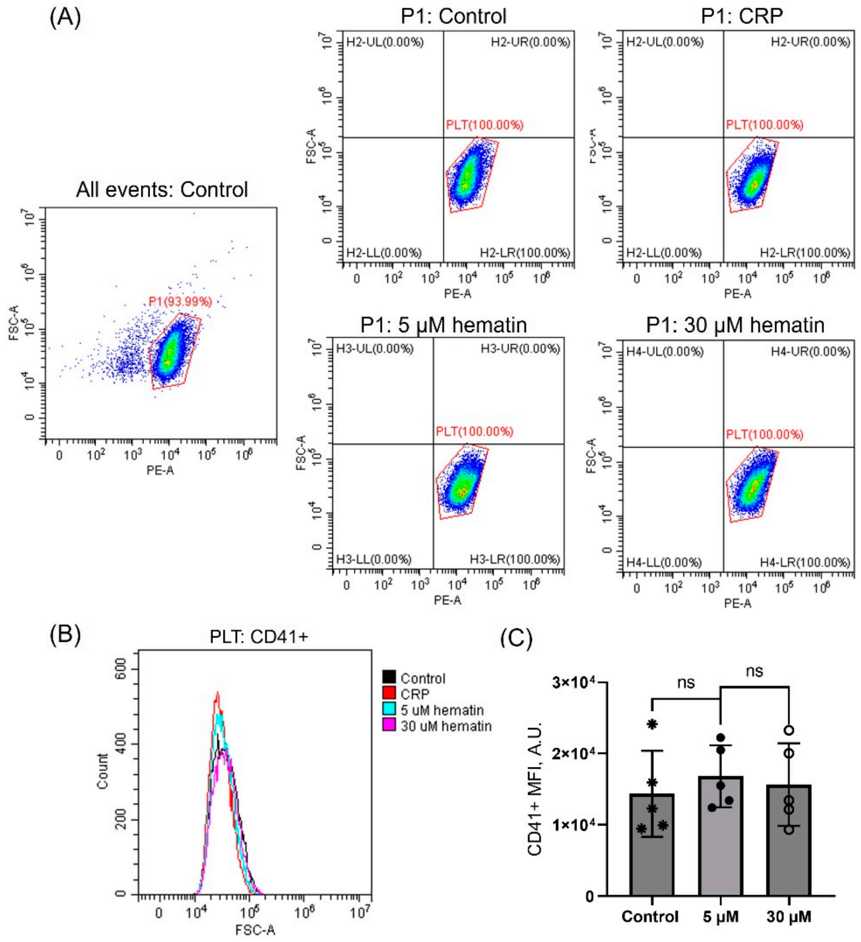


**Figure S5. Hematin administration did not affect platelet count.** Washed platelets (2 x $10^7$ cells/mL) were incubated with either CRP (0.5 μg/ml, 5 min, 37°C) or hematin at indicated concentrations. Then, the CD41 antibody (1:30, 10 min, 37°C) were added to the suspension with further analysis by flow cytometry at FL2. (A) Histograms of event distribution by count and CD41+ MFI. (A, B) Flow cytometry density plots and overlay histograms. (C) Quantification of the data presented in (B). One-way ANOVA, Dunnet's test, ns – not significant.

## S6. Hematin-induced platelet activation is independent of ADP and TxA2 signaling

Hemin-induced platelet activation was not affected by classical platelet inhibitors such as the cyclooxygenase inhibitor (indomethacin) or the $P2Y_{12}$ inhibitor (cangrelor)[4]. Using the original laser diffraction method, we also demonstrated that either $P2Y_{12}$ (ARC, 10 nM) or thromboxane prostanoid (TP, SQ29548, 1 μM) receptor inhibition does not affect hematin-induced platelet aggregation (Figure S5). It should be mentioned that PRP was used for the analysis of ADP-triggered transformations, and therefore, the working hematin concentration was adjusted accordingly to 150 μM to overcome the capacity of the plasma protein scavenging system (Figure S5A).

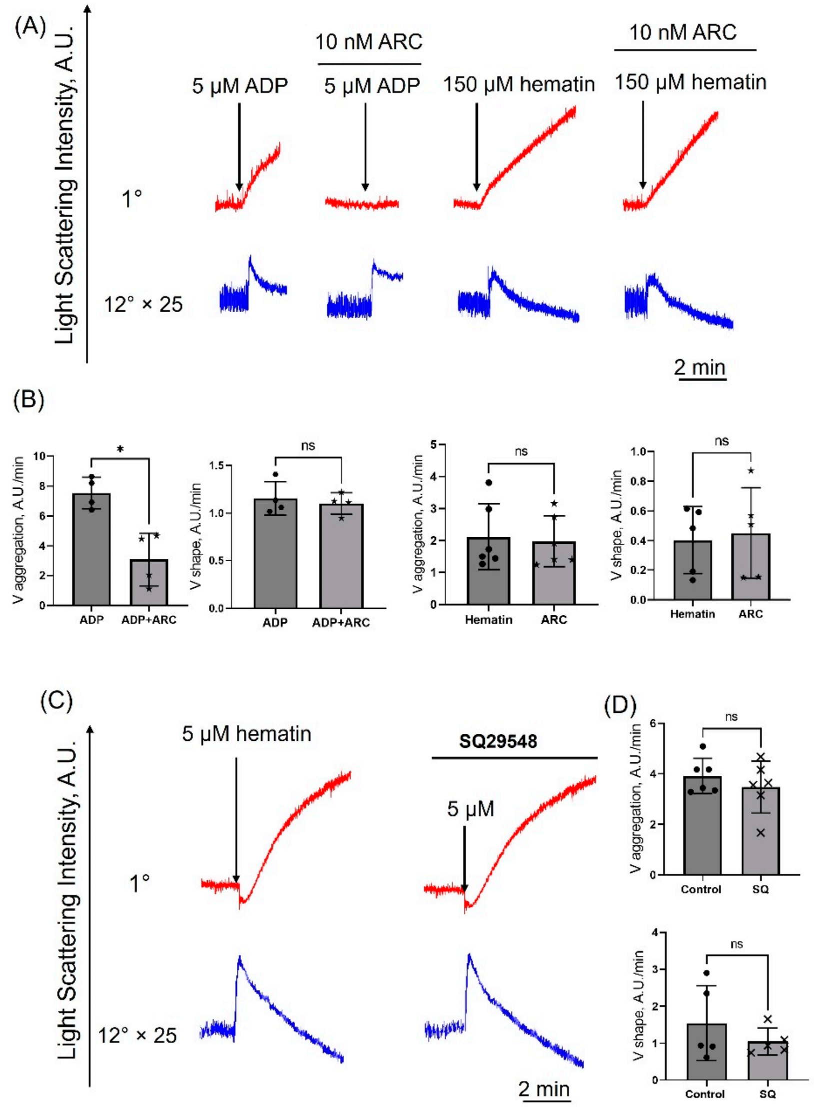


**Figure S6. Hematin-induced platelet activation is independent of ADP and TxA2 signaling.** PRP (A) or washed platelets (C) were suspended in HEPES buffer (1.5 mM $Ca^{2+}$) in the cuvette with continuous stirring (1200 rpm) at 37°. Hematin at indicated concentrations was added to PRP or platelet suspension (2 x $10^7$ cells/mL) corresponding to control, and aggregation (1°) and shape change (12°) were registered. ARC (10 nM) or SQ29548 (1 µM) were incubated with PRP or platelets for 5 min, and then hematin was added to the cell suspension. For the ADP experiments, PRP was used; therefore, to overcome the protective effect of albumin traces, hematin concentration was increased 5-fold. (A) Representative curves from one of six experiments using the LaSca laser analyzer. (B) Quantification of the data presented in (A). (C) Representative curves from one of six experiments using the LaSca laser analyzer. (D) Quantification of the data presented in (C). Mann-Whitney U-test, *, p<0.05, ns – not significant.

## S7. Original dot plots for Flow Cytometry experiments.

**Dot plots for Figure 6 (C-AM)**

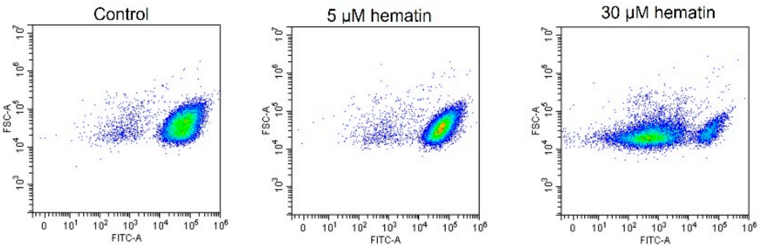


**Dot plots for Figure 7 (Annexin-V)**

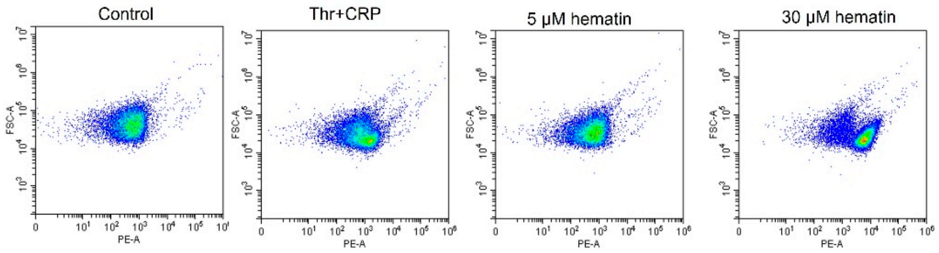


**Dot plots for Figure 8 (CD62P)**

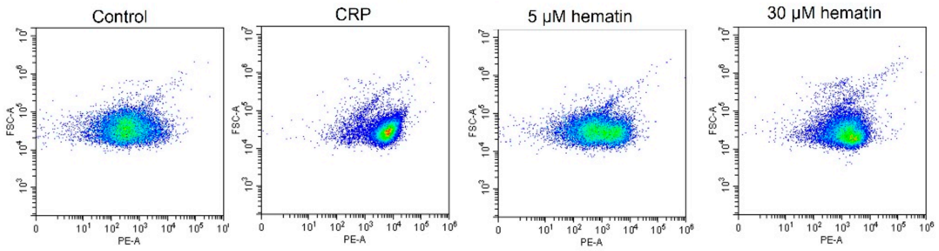


**Dot plots for Figure 8 (Mepacrine)**

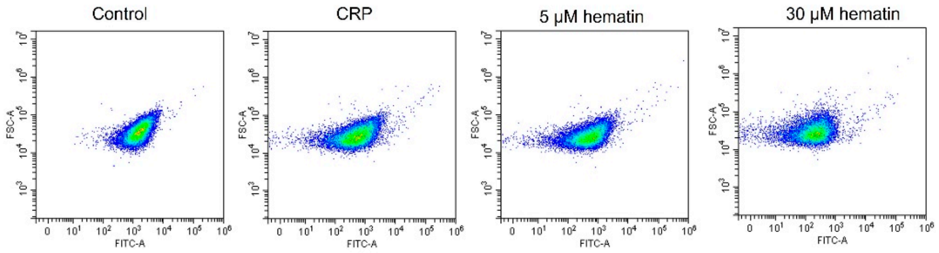


**Dot plots for Figure 9 (Fibrinogen)**

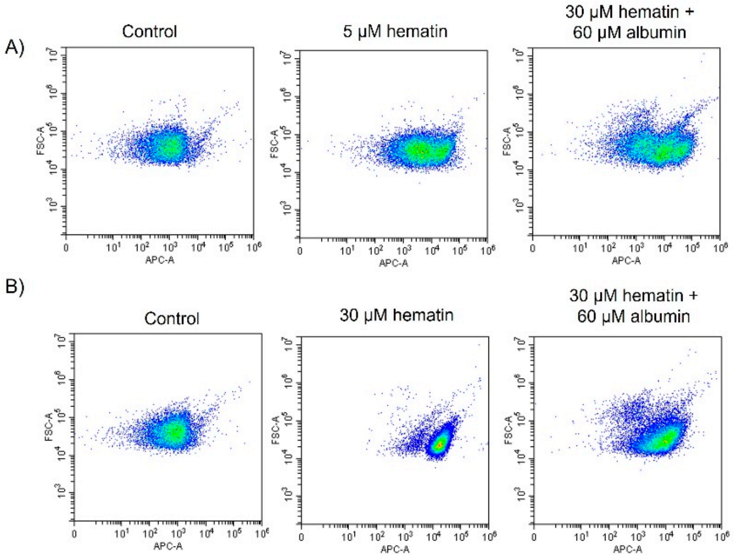


**Figure S7. Original dot plots for Flow Cytometry experiments.**

**References:**


1. Mindukshev I, Fock E, Dobrylko I, Sudnitsyna J, Gambaryan S, Panteleev MA: Platelet Hemostasis Reactions at Different Temperatures Correlate with Intracellular Calcium Concentration. International Journal of Molecular Sciences 2022, 23(18):10667.
2. NaveenKumar SK, SharathBabu BN, Hemshekhar M, Kemparaju K, Girish KS, Mugesh G: The Role of Reactive Oxygen Species and Ferroptosis in Heme-Mediated Activation of Human Platelets. ACS Chemical Biology 2018, 13(8):1996-2002.
3. Ponomareva AA, Nevzorova TA, Mordakhanova ER, Andrianova IA, Rauova L, Litvinov RI, Weisel JW: Intracellular origin and ultrastructure of platelet-derived microparticles. J Thromb Haemost 2017, 15(8):1655-1667.
4. Bourne JH, Colicchia M, Di Y, Martin E, Slater A, Roumenina LT, Dimitrov JD, Watson SP, Rayes J: Heme induces human and mouse platelet activation through C-type-lectin-like receptor-2. Haematologica 2021, 106(2):626-629.
